# Pattern of cephalosporin and carbapenem-resistant *Pseudomonas aeruginosa*: a retrospective analysis

**DOI:** 10.1016/j.ijregi.2023.11.012

**Published:** 2023-11-19

**Authors:** Salma AlBahrani, Thikrayat Qazih Alqazih, Ali Ahmad Aseeri, Reem Al Argan, Dania Alkhafaji, Nora Abdullah Alrqyai, Sami Mohamed Alanazi, Dima Saleh Aldakheel, Qassim Hassan Ghazwani, Salah Saeed Jalalah, Anwar Khalid Alshuaibi, Hanadi Ali Hazzazi, Jaffar A. Al-Tawfiq

**Affiliations:** 1 Infectious Disease Unit, Specialty Internal Medicine, King Fahd Military Medical Complex, Dhahran, College of medicine-Imam Abdulrahaman Bin Faisal University, Dammam, Saudi Arabia; 2 Microbiology Department, King Fahd Military Medical Complex, Dhahran, Saudi Arabia; 3 Department of Internal Medicine, College of Medicine, Imam Abdulrhaman Bin Faisal University, King Fahd Hospital of the University, Khobar, Saudi Arabia; 4 Infection control Department, King Fahd Military Medical Complex, Dhahran, Saudi Arabia; 5 Infectious Disease Unit, Specialty Internal Medicine, Johns Hopkins Aramco Healthcare, Dhahran, Saudi Arabia; 6 Infectious Disease Division, Department of Medicine, Indiana University School of Medicine, Indianapolis, USA; 7 Infectious Disease Division, Department of Medicine, Johns Hopkins University School of Medicine, Baltimore, USA

**Keywords:** *Pseudomonas aeruginosa*, MDR, Colistin, CRE, carbapenemase

## Abstract

**Objectives:**

Despite its financial cost on the world's health care system, *Pseudomonas aeruginosa* antibiotic resistance has been increasing. Therefore, the goal of this study was to assess the level of antimicrobial resistance to anti-pseudomonas medicines, specifically β-lactam medications such as cephalosporin and carbapenems. In addition, we evaluate the prevalence of multi-drug resistance to *P. aeruginosa*, particularly during the years of the COVID-19 pandemic.

**Methods:**

This retrospective analysis covered the period from January 2019 to December 2022 and included cephalosporin- and carbapenem-resistant *P. aeruginosa* isolates. The real-time polymerase chain reaction Genexpert test (CARBA-R kit) was used for the detection of genes responsible for carbapenemase resistance.

**Results:**

During the time of the study, 1815 clinical isolates of *P. aeruginosa* were identified and 160 (9%) were resistant to carbapenems and cephalosporins. The resistance rates were 32.5% (13/597) in 2019, 11.2% (44/393) in 2020, 7% (26/369) in 2021, and 11% (50/456) in 2022. Of those isolates, multidrug-resistant rates were 6.7%, 86.3%, 57.7%, and 56%, per year over the study period. Using Genexpert test, 88 (93.6%) of multidrug-resistant *P. aeruginosa* were negative for carbapenemase genes.

**Conclusion:**

This study emphasizes the alarming patterns of carbapenem and cephalosporin resistance among *P. aeruginosa* clinical isolates. Furhter surviellance from different centers and different regions is required.

## Highlights

• 1815 clinical isolates were identified
• 160 (9%) were resistant to carbapenems and cephalosporins
• 93.6% of multidrug-resistant *Pseudomonas aeruginosa* were negative for carbapenemase genes
• The most common resistance was among blood isolates (*P*-value < 0.00001)

## Introduction

Global public health is seriously threatened by antibiotic resistance, which also raises morbidity, mortality, and medical expenses. An important bacterium recognized for its capacity to acquire resistance to several antimicrobial agents is *Pseudomonas aeruginosa*. Since carbapenems are thought to be the last line of defense against *P. aeruginosa*, the advent of carbapenem-resistant *P. aeruginosa* has recently confounded treatment choices [1], [2], [3]. *P. aeruginosa* is one of the most virulent organisms and is frequently the leading cause of nosocomial infections including ventilator-associated pneumonia (VAP), catheter-associated urinary tract infections (CAUTI), surgical site infections (SSI), and central line associated blood stream infections (CLABSI), with an associated higher morbidity and mortality [4]. Optimizing treatment plans and infection prevention practices requires a thorough understanding of the prevalence and patterns of resistance of *P. aeruginosa*.

There are multiple studies reporting increase in the rate of antibiotic resistance in *P. aeruginosa*, in particular to β-lactams, aminoglycosides, and fluoroquinolones in many areas of the world [5]. *P. aeruginosa* is the most commonly isolated nosocomial bacteria in Saudi Arabia hospitals among other gram-negative organisms [6]. Concerning levels of antibiotic resistance have been found in *P. aeruginosa* isolates in Saudi Arabia, according to multiple studies. For instance, *P. aeruginosa* isolates from diverse clinical specimens in a tertiary care hospital in Riyadh, Saudi Arabia, showed a high rate of carbapenem resistance of 34% in 2004 and 74% in 2009 [7]. An additional study evaluated the susceptibility pattern of inpatient and outpatient isolates of *P. aeruginosa* in a Saudi hospital over a 6-year period (1998-2003). The study showed the following resistance rates of inpatient isolates: piperacillin (3.5% and 16%), ceftazidime (6% and 12.3%), imipenem-cilastatin (1.4% and 11%), and ciprofloxacin (2.3% and 10.7%), in 1998 and 2003, respectively [8]. A recent study from the same hospital showed increasing resistance rates of 8-19% and 18-27% for ceftazidime and imipenem-cilastatin, respectively over the study period 2013-2018 [9]. Management of infectious diseases has become more difficult as a result of the COVID-19 pandemic, including the change in the patterns of antibiotic resistance. Increasing use of antibiotics during the pandemic, both for the treatment of COVID-19 patients initially and as a preumptive therapy in seriously ill patients, may have influenced the development and spread of antibiotic resistant organisms, including *P. aeruginosa*. Thus, inappropriate antibiotic prescription during the COVID-19 has exacerbated another serious public health catastrophe [10], [11], [12]. These findings underline how critical it is to keep an eye on and treat antibiotic resistance in *P. aeruginosa* infections globally, especially during the COVID-19 pandemic. To prevent the spread of multidrug-resistant *P. aeruginosa* strains, infection control measures and antibiotic stewardship can all be improved by having a thorough understanding of local resistance patterns and trends and the prevalence of carbapenemase genes. This retrospective study aims to evaluate the extent of antimicrobial resistance in *P. aeruginosa* isolates throughout the COVID-19 pandemic years, particularly to β-lactam drugs such as cephalosporins and carbapenems. The study also accounted for the prevalence of carbapenemase genes in *P. aeruginosa* .

## Materials and method

From January 2019 to December 2022, this retrospective study was carried out at the King Fahad Military Medical Complex (KFMMC) microbiology laboratory in Dhahran, Saudi Arabia. As a tertiary care hospital, KFMMC has 335 beds total, including 24 ICU beds. Blood, sputum, tracheal aspirate, urine, and wounds were among the body sites from which *P. aeruginosa* isolates were examined. Samples were taken from patients in the inpatient wards (intensive care units and geenral hospital units) and outpatient clinics (primary care and specialty clinics).

### Pseudomonas identification and susceptibility

Using the BD Phoenix Automated identification and Susceptibility System, *P. aeruginosa* isolates were identified to the *s*pecies level. The Clinical and Laboratory Standards Institute (CLSI) breakpoints provided in the BD Phoenix Update Disk Version V7.01A were used to interpret antibacterial susceptibilities. The study concentrated on the susceptibility of *P. aeruginosa* to carbapenems (imipenem and meropenem) and cephalosporins (cefepime and ceftazidime). Multidrug-resistant (MDR) isolates were those that exhibited non-susceptibility to at least one agent in three antimicrobial categories (aminoglycosides, quinolones, and piperacillin/tazobactam), as well as being resistant to both cephalosporins and carbapenems [13].

### Colistin broth microdilution test

The Compact Antimicrobial Susceptibility Panel (ComASP) broth microdilution test was used to determine the minimum inhibitory concentration (MIC) for Colistin. The test was conducted in accordance with the manufacturer's instructions, and the CLSI guidelines were used to determine the MIC breakpoints. Briefly, a bacterial suspension that is equivalent to standardization of a 0.5 McFarland was prepared and then diluted in saline to reach 1:20 suspension. Subsequently, 400 μl of that suspension was added to a Muller Hinton broth, then 100 μl was transferred to microtitration plate that contained seven wells of two-fold dilution concentrations (0.25-16 μg/ml) of colistin. Finally, the microtitration plate was then incubated overnight at 37°C, and the turbidity was checked visually for the determination of MIC breakpoint according to CLSI guidelines.

### Carbapenemase gene molecular detection

MDR *P. aeruginosa* isolates were tested for possessing carbapenemase genes using the Cepheid GenXpert instrument Version 4.8. The GenXpert CARPA-R kit uses polymerase chain reaction technique through multiple thermal cycling during DNA extraction and carbapenemase gene sequence amplification. The following genes were examined: *Klebsiella pneumoniae* carbapenemase (blaKPC), New Delhi metallo-beta-lactamase (blaNDM), Verona integron-mediated metallo-beta-lactamase (blaVIM), oxacillinase-48 (blaOXA-48), or imipenemase (blaIMP).

### Statistical analysis

In order to do the statistical analysis, Microsoft Office Excel was used. Charts were used as appropriate to illustrate the data, and variables were reported as numbers and percentages. Comparisons between different groups was done using X-square and a *P*-value of <0.05 was considered significant.

## Results

**Figure 1:**
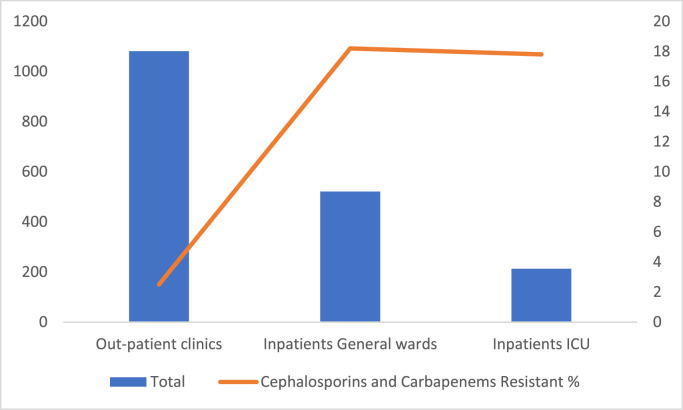
Distribution of cephalosporins and carbapenems resistant *Pseudomonas aeruginosa* cases per location. ICU, intensive care unit. The bar represents the number of isolates (Left Y-axis) and the line represents the percentage of resistance (left Y-axis).

**Figure 2:**
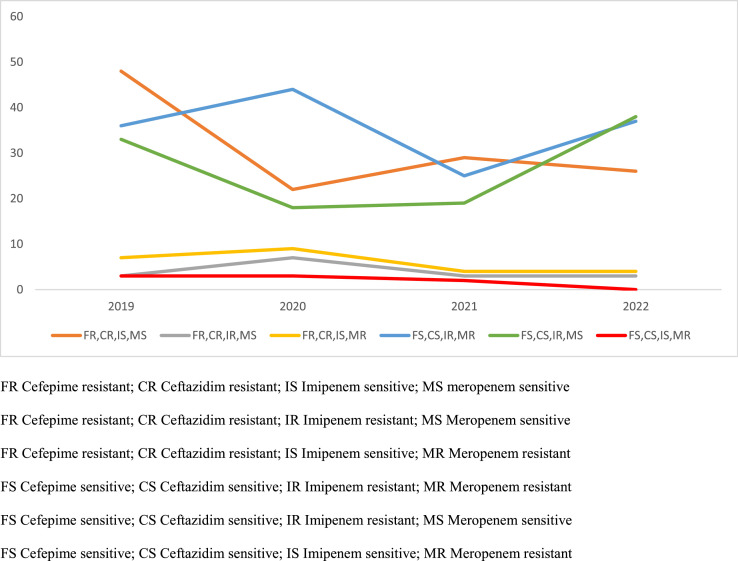
A line chart showing the frequency of antimicrobial resistance phenotypes of *Pseudomonas aeruginosa* over the study period. CR, ceftazidim resistant; CS, ceftazidim sensitive; FR, cefepime resistant; FS, cefepime sensitive; IR, imipenem resistant; IS, imipenem sensitive; MS, meropenem sensitive; MR, meropenem resistant

During the study period, a total of 1815 clinical isolates of *P. aeruginosa* were identified during the study period. And of those 160 (9%) were cephalosporins and carbapenems resistant organisms. Of those 94 (58.7%) were MDR (Table 1). Of the 597 *P. aeruginosa* in 2019, 40 (6.7%) were resistant to carbapenems and cephalosporins and 13 (32.5%) of these were MDR. There were 393 *P. aeruginosa* in 2020, 44 (11.2%) exhibited cephalosporin and carbapenem resistance and 38 (86.3%) of the resistant isolates were MDR. Of the 369 isolates of *P. aeruginosa* in 2021, 26 (7%) were resistant to cephalosporins and carbapenems and 15 (57.7%) of these were MDR. Of the 456 *P. aeruginosa* in 2022, 50 (11%) exhibited cephalosporin and carbapenem resistance, and 28 (56%) of those were MDR. There was a significant difference in the percentage between inpatient and intensive care isolates compared to outpatient isolates (*P*-value is <0.00001) (Figure 1). The most common phynotypes were: cefepime sensitive, ceftazidim sensitive, imipenem resistant, and meropenem sensitive followed by cefepime sensitive, ceftazidim sensitive, imipenem sensitive and meropenem resistant (Figure 2). Only one (1%) of the MDR *P. aeruginosa* were colistin resistant.

**Table 1:** Distribution of cephalosporins and carbapenems resistant *Pseudomonas aeruginosa* over the study period.

Year	Total number of *Pseudomonas aeruginosa* isolates	Cephalosporins and carbapenems resistant Pseudomonas; number (%)	Multidrug-resistant isolates among the cephalosporins and carbapenems resistant Pseudomonas cases; number (%)
2019	597	40 (6.7)	13 (32.5)
2020	393	44 (11.2)	38 (86.3)
2021	369	26 (7)	15 (57.7)
2022	456	50 (11)	28 (56)
All years	1815	160 (9)	94 (58.7)

Of the 42 *P. aeruginosa* isolates from blood samples, eight (19%) were resistant to cephalosporins and carbapenems compared to 26 (3.5%) of the 750 the urine isolates. In 588 *P. aeruginosa* isolates of respiratory samples, 73 (12.4%) showed resistance to cephalosporins and carbapenems compared to 39 (15.3%) of 255 of the isolates from wound samples (Table 2). The most common resistance was among blood (19%) compared to other sites (*P*-value was <0.00001) (Table 2).

**Table 2:** Distribution of cephalosporins and carbapenems resistant *Pseudomonas aeruginosa* cases by body sites.

Body site	Total pseudomonal isolates	Cephalosporins and carbapenems resistant Pseudomonas number (%)
Blood	42	8 (19)
Urine	750	26 (3.5)
Respiratory	588	73 (12.4)
Wound	255	39 (15.3)
Ear	180	14 (7.8)
All	1815	160 (8.8)

Among the 160 cephalosporins and carbapenems resistant *P. aeruginosa*, 94 (59%) were MDR. Using Genexpert CARBA-R kit test, 88 (93.6%) of MDR *P. aeruginosa* were negative for carbapenemases genes . However, there were three (3.2%) NDM two (2.1%) IMP, and one (1%) VIM (Table 3).

**Table 3:** The prevalence of carbapenemase enzymes (sing Genexpert CARBA-R kit test) and colistin resistance among MDR *Pseudomonas aeruginosa* isolates.

			Number (%) of *Pseudomonas aeruginosa* MDR Isolates with the specified test result				
Year	Number of cephalosporins and carbapenems resistant Pseudomonas cases	Number of MDR isolates among the cephalosporins and carbapenems resistant Pseudomonas cases	Negative polymerase chain reaction	New Delhi metallo	Number imipenemase	verona integron-mediated metallo	colistin resistant
2019	40	13 (32.5)	13 (100)	0	0	0	0
2020	44	38 (86.3)	37 (97.4)	0	0	1 (2.6)	0
2021	26	15 (57.7)	12 (80)	2 (13.3)	1 (6.7)	0	0
2022	50	28 (56)	26 (93)	1 (3.5)	1 (3.5)	0	1 (3.6)
All	160	94 (58.7)	88 (93.6)	3 (3.2)	2 (2.1)	1 (1)	1 (1)

MDR, multidrug-resistant.

## Discussion

This study shows that cephalosporin and carbapenem resistance is a prevalent occurence among *P. aeruginosa* isolates. The rates of resistance are consistent with earlier studies done in healthcare settings, which also found considerable levels of resistance and is consistent with observations from other regions [14]. In our study, the prevalence of *P. aeruginosa* resistance to cephalosporins and carbapenems fluctuated between 6.7% and 11.2% over the course of the study. Recent studies have focused on how the COVID-19 pandemic has impacted antimicrobial resistance. Coseriu et al. observed changes in *P. aeruginosa* susceptibility to carbapenems, piperacillin/tazobactam, and amikacin before and throughout the COVID-19 pandemic (2017-2022) with lower rates of resistance to carbapenems and fluoroquinolones during the pandemic as a result of the adequate dissemination of antibiotic therapeutic guidelines [15]. However, a different study found that antibiotic resistance pf *P. aeruginosa* increased during the pandemic period compared to the pre-pandemic period [16]. It is important to note that 6.4% of the MDR *P. aeruginosa* had detectable carbapenemase genes using GenXpert CARPA-R kit. The NDM, IMP, and VIM carbapenemase genes, respectively, were present in three (3.2%), two (2.1%), and one (1%) of the isolates. These findings are in line with prior studies of carbapenemase-producing *P. aeruginosa* [17]. The absence of carbapenemase genes does not rule out the possibility of other resistance mechanisms, and it is crucial to note given that *Pseudomonas* can acquire resistance through a variety of genetic mechanisms, including mutations in target sites and activation of efflux pumps [18]. Only one (1%) of the MDR *P. aeruginosa* were colistin resistant. This finding is close to 3.1% resistance reported in one study from Saudi Arabia [19]. Although, the majority of MDR *P. aeruginosa* lacked detectable carbapenemase genes, a small proportion tested positive for NDM, IMP, or VIM carbapenemase genes. This highlights the significance of meticulous surveillance and infection control techniques as a means of preventing the emergence of resistant strains by illuminating the complex nature of Pseudomonas’ resistance mechanisms. However, the fact that only one hospital was included in the study is a limitation of the study, and the findings might not be applicable to other institutions. Another limitation is the retrospective nature and not including the demographics and outcome of the included patients. Thus, furhter surviellance from different centers and different regions is required to further charcaterize the evolution of the resistance pattern on *P. aeruginosa.*

## Declarations of Competing Interest

The authors have no competing interests to declare.
